# Effect of Diisocyanates as Compatibilizer on the Properties of BF/PBAT Composites by In Situ Reactive Compatibilization, Crosslinking and Chain Extension

**DOI:** 10.3390/ma13030806

**Published:** 2020-02-10

**Authors:** Xiwei Xie, Caili Zhang, Yunxuan Weng, Xiaoqian Diao, Xinyu Song

**Affiliations:** 1 School of Materials and Mechanical Engineering, Beijing Technology& Business University, Beijing 100048, China; 18931729309@163.com; 2 Beijing Key Laboratory of Quality Evaluation Technology for Hygiene and Safety of Plastics, Beijing Technology and Business University, Beijing 100048, China; diaoxiaoqian@btbu.edu.cn (X.D.); songxinyu722@163.com (X.S.)

**Keywords:** biodegradable composites, PBAT, diphenylmethane diisocyanate, compatibilizer, impact strength

## Abstract

Due to the hydrophobic nature of poly (butylene terephthalate) (PBAT), and the hydrophilic nature of bamboo flour (BF), a BF/PBAT (50/50) blend shows low mechanical properties, and especially shows poor impact strength. In order to increase the interfacial adhesion between BF and PBAT, diisocyanate was used as a reactive compatibilizer to modify bamboo powder. A series of BF/PBAT composites were prepared by the method of mixing and melting in an internal mixer. After adding reactive compatibilizer 4,4′-methylenebis(phenyl isocyanate) (MDI), BF/PBAT (50/50) composites with high mechanical properties were successfully prepared. The tensile strength, elongation at break, and impact strength of the BF/MDI-2/PBAT composite with 2 wt % MDI content were increased by 1.9, 6.8, and 4.3 times respectively over the BF/PBAT blend without the added MDI. The higher toughening effect of MDI in BF/PBAT composites can be mainly ascribed to the improved interface bonding between BF and PBAT. The isocyanate group of MDI can react with the hydroxyl group on the BF surface and in situ formation of the carbamate group on the BF surface. The residual isocyanate can then react with the hydroxyl group of PBAT and form carbamate groups. The rheological behaviors demonstrate that addition of appropriate amounts of MDI, 1 wt % and 2 wt %, can promote the flowability of the molten BF/PBAT composites due to the decrease in interparticle interaction between bamboo powder and the increase in the thermal motion of the molecules.

## 1. Introduction

Natural fibers have attracted much attention due to their renewability and complete degradation or composting in the soil without any toxic or harmful gases produced. In particular, natural fibers reinforced with plastic composites have high mechanical properties, and have great potential in the application of packaging materials [1]. Previous works have confirmed that blending plastic with appropriate sisal, bagasse, bamboo flour (BF), flax, wood powder, or other cellulose fibers could significantly reinforce the mechanical properties of the plastic composite, especially the bending modulus and impact strength [2,3,4,5,6,7]. The physical properties, such as length, diameter, growth cycle, and cost, of the most-used natural fibers are listed in Table 1. When taking into account of the cost of production and growth cycle, bamboo flour fiber is considered as the most suitable renewable fillers. Bamboo fiber is composed of cellulose, lignin, hemicellulose, and other macromolecules, which have strong rigidity. In addition, it has the advantages of antibacterial, low cost, and high recovery rate. More significantly, bamboo flour can not only be used as a reinforcement, but also can act as a nucleation agent to improve the crystallization performance and crystallization rate of the composite [3,7,8,9].

Biodegradable polyesters such as poly (butylene adipate/terephthalate) (PBAT), poly (e-caprolactone) (PCL), and poly (L-lactide) (PLA) are widely used in many areas. In particular, PBAT shows good toughness, and high elongation at break, which can be used to produce blown-film products. However, due to the high cost and its limited strength, PBAT has almost no applications in injection products. Therefore, the combination of PBAT with bamboo flour could be an effective way to improve the performance at a relatively low cost. 

The reinforcing effect of bamboo powder for a polymer matrix mainly depends on the particle size and interface bonding with the matrix. While the interface plays a critical role in determining the mechanical properties, such as transfer stress and distribution bond [12], it has been found that the poor dispersion, weak interfacial adhesion, and ultimately inferior composite quality of the bamboo-powder-reinforced composites mainly come from the physical and chemical incompatibility between the fiber and the matrix [13,14,15]. It was reported that various modification methods aiming at overcoming the incompatibility and refining the interfacial adhesion between polymer and fiber, include chemical modification of bamboo-powder surface or the addition of appropriate compatibilizer, and the latter was the most effective way to overcome two-phase incompatibility [12,16,17,18,19]. The most used in situ reactive compatibilizers to compatibilize PBAT and natural fibers contain epoxy or diisocyanate groups, which could easily form chemical bonds between both PBAT and natural fiber [20,21,22,23]. 

Due to the existence of active NCO groups, diisocyanate was widely used as a reactive compatibilizer [24]. A previous report indicates that diisocyanate has a positive effect on compatibilizing bamboo flour/plastic composites [25]. Saengon et al. modified starch nanocrystals with lysine diisocyanate, esterified the fillers, solved the problem of hydrophobicity of fillers in phosphate buffer solution (PBS), and made the fillers have better dispersion in PBS. When 1 wt % compatibilizer was added, the elongation at break increased by 51.1% and the PBS was effectively strengthened [26]. Chan et al. studied the compatibilizing system with PHBV (poly-hydroxybutyrate-co-hydroxyvalerate) and wood powder as the main substrates. It was found that when the content of 4, 4-methylenebis (phenyldiisocyanate) (MDI) was 2 wt %, the tensile strength of the composite increased by 256%, but when the content was 4 wt %, it returned to the original state [27].

In this work, in order to increase the interfacial adhesion between BF/PBAT composites, we selected 4,4′-methylenebis(phenyl isocyanate) (MDI) as a reactive compatibilizer to modify the bamboo-flour surface. A series of BF/PBAT composites were prepared by the method of mixing and melting in an internal mixer. After adding a reactive compatibilizer, BF/PBAT (50/50) composites with high mechanical properties were successfully prepared. The morphology of the fracture surface and rheology behavior of the composites were fully characterized.

## 2. Experimental

### 2.1. Materials

Bamboo flour (BF) with the mesh number of 150 was provided by Mujiang Weihua Flavor Factory, Shuangshui Town, Xinhui District, Jiangmen City, China. Before mixing, bamboo powder was first dispersed in ethanol solution to remove impurities and soil residues from the surface, then filtered and dried in an air circulation oven at 80 °C for 24 h. PBAT, in which the mass fraction of BT is 43.5 wt %, while that of BA is 56.5 wt % by measuring (FS-0330, melt index 4 g/10 min, density 1.26 g/cm^3^), was purchased from Jinhuizhaolong High Tech Co., Ltd., (Xiao Yi, China). 4, 4-methylenebis (phenyldiisocyanate) (MDI) (>98%) was obtained from J&K Scientific Ltd, Chengdu, China and used as received. Reagent grade ethanol was purchased from Beijing Chemical Reagent Factory, Beijing, China, and used as received. 

### 2.2. Sample Preparation

#### 2.2.1. Purification of Bamboo Flour

First, disperse the bamboo powder in the ethanol solution on a round-bottomed flask with strong mechanical stirring for 2 h. After that, put the flask on an ultrasonic vibration instrument for 30 min. Then filter and dry the bamboo flour at 80 °C for 6–8 h until the constant weight is reached.

#### 2.2.2. Preparation of Composites 

Bamboo powder, PBAT, and diisocyanate were mixed with a certain proportion in an internal mixer to prepare the composite materials. The mixing temperature was controlled at 178 °C, and the mixing proportion was as listed in Table 2. In order to obtain better uniformity and dispersion, PBAT (50 wt %) and bamboo powder (50 wt %) were premixed in a high-speed mixer at 80 rmp for 3 min. Then, the BF/PBAT blend and the coupling agent were mixed in the mixer for 5 min. The mixture was smashed and dried in an oven at 80 °C for at least 24 h before injection molding. Finally, tensile and impact splines were made and ready for testing.

### 2.3. Characterization 

Attenuated total reflection Fourier transform infrared (ATR-FTIR) spectra of composites were monitored using a Nicolet iS50 FT-IR spectrometer (Thermos Fisher scientific, Waltham, MA, USA) in a wavenumber range of 4000–400 cm^−1^. Thermogravimetric analysis (TGA) was carried out on a (Hitachi, Tokyo, Japan) Instruments STA7200 instrument in a temperature range of 30 to 600 °C with a heating rate of 10 °C/min under nitrogen atmosphere. 

A microcomputer-controlled electronic universal testing machine (Mester Industrial System (China) Co., Ltd., Jinan, China) was used to test the tensile strength of the sample (1500 mm × 10 mm × 4 mm) made according to the test standard ASTM D638 [28] in the laboratory environment for 24 h, and the tensile speed was 50 mm/min. Each group of specimens was tested more than five times, and the test results were averaged.

The impact sample is a standard spline (800 mm × 10 mm × 4 mm), which is notched according to the standard ASTM D256 [29]. After the spline was placed in the laboratory environment for 24 h, the impact test was carried out with a 5J pendulum by using an electronic cantilever impact tester (Chengde Jinjian Testing Instrument Co., Ltd., Chengde, China). Each group of splines was tested more than five times, and the test results were averaged.

The rheological properties of the composites were measured with a rotational rheometer (Anton Paar MCR-502, Anton PA Trading Co., Ltd., Shanghai, China). The viscosity, storage modulus, and loss modulus of the composites at 180 °C in the range of 1–100 rad/s were measured.

The morphologies of the impact-fractured surfaces of BF/PBAT composites were observed by scanning electron microscopy (SEM, S-4800, Hitachi, Tokyo, Japan) at an accelerating voltage of 10 kV in SE mode. All samples were sputtered with a thin layer of gold prior to SEM observation.

## 3. Results and Discussion

### 3.1. Mechanical Properties of the Composites

**Figure 1:**
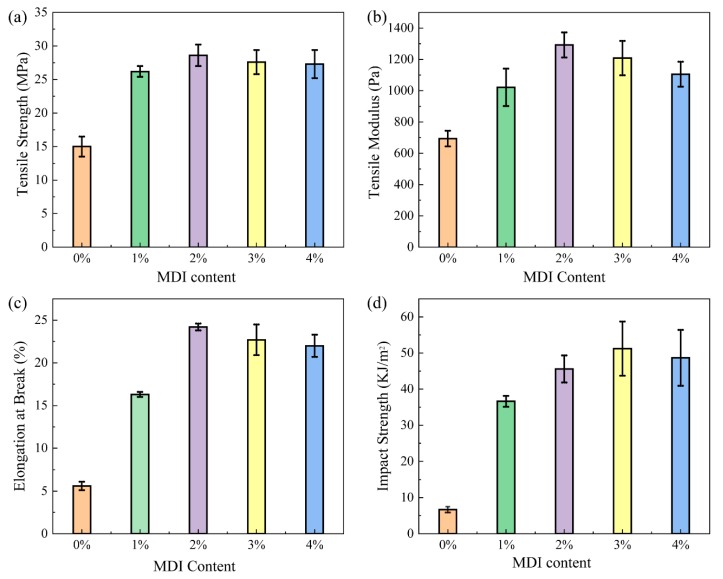
Effect of 4,4-methylenebis (phenyldiisocyanate) (MDI) contents on the mechanical properties of BF/PBAT composite: (**a**) tensile strength, (**b**) tensile modulus, (**c**) elongation at break, and (**d**) impact strength.

The mechanical properties of BF/PBAT composites without compatibilizer is unsatisfactory due to the poor interfacial compatibility between bamboo powder and PBAT, because of their polarity differences. Impact strength and elongation at break can indicate the fracture resistance of the composite under the action of high-speed stress, which is mainly related to the structure of matrix and filler, the degree of two-phase bonding and the difficulty of filler pulling out from the matrix. The tensile strength and modulus indicate the tensile properties of the composite during the failure process. The tensile strength, tensile modulus, impact strength, and elongation at break of the BF/PBAT composites without and with different amounts of compatibilizer are listed in Table 3 and also shown in Figure 1. 

From Figure 1a, the tensile strength of BF/PBAT (50/50) blend without MDI is 15 MPa. With the addition of MDI content increase, the tensile strength of the composites was first increased and then decreased, and finally leveled off. It was found that the BF/MDI-2/PBAT showed higher tensile strength than that of other composites. The changing trends of tensile modulus and elongation at break of the composites presented in Figure 1b,c are in agreement with the tensile strength showed in Figure 1a, and BF/MDI-2/PBAT shows the highest value. Considering the measurement error, the mechanical properties of the composites can be greatly improved by 2 wt %–3 wt % loadings of diisocyanate. Impact strength for the composites without and with different content of MDI are presented in Figure 1d. It could be found that MDI can significantly enhance the impact strength of BF/PBAT composites, and the impact strength of BF/MDI-3/PBAT shows an increase of approximately seven times higher than that of BF/PBAT composites without MDI.

The above results showed that the addition of MDI greatly improved the mechanical properties of the BF/PBAT composites. If without MDI, the gaps between the PBAT matrix and the bamboo powder acted as stress concentrators in the composite, which induced cracks upon tensile testing that resulted in lower mechanical properties. The previously work reported that diisocyanate was a good coupling agent and can be used to modify wood–plastic composite [25,30,31]. In this study, diisocyanate was selected as compatibilizer to improve the interface bonding between BF and PBAT. The isocyanate group of diisocyanate reacted with the hydroxyl group of the BF surface, and in situ formation of the carbamate group on the BF surface. The residual isocyanate can then react with the hydroxyl and carboxyl end group of PBAT and form urethane bonds. According to the Table 1 and Figure 1, when the diisocyanate content is 2 wt %–3 wt %, the mechanical properties of the composite are significantly improved. The impact strength and elongation at break are increased most obviously. That is because diisocyanate can also act as the chain extender of PBAT in processing, increasing the length and molecular weight of the molecular chain, so its toughness is significantly improved.

### 3.2. Structural Characterization 

**Figure 2:**
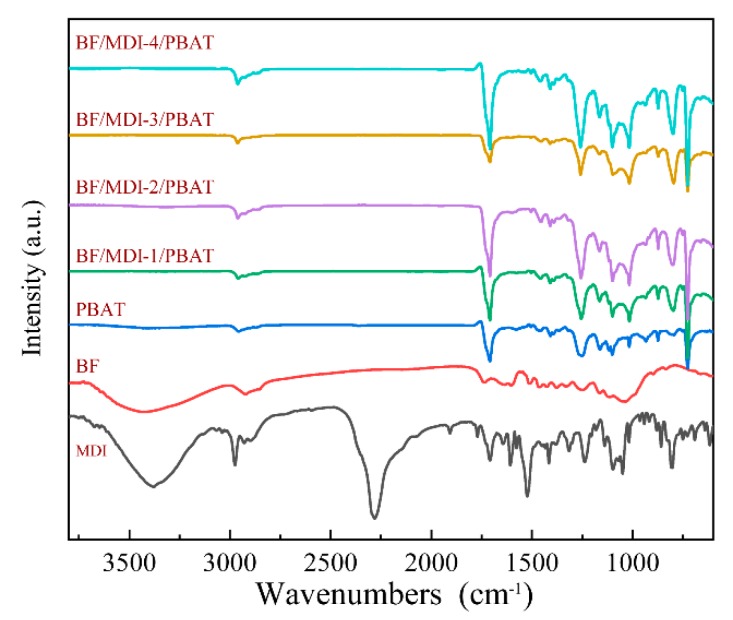
ATR spectra of pure PBAT, BF, MDI, and BF/PBAT composites.

The ATR spectra of pure PBAT, BF, MDI, and the BF/PBAT composites with different contents of MDI are shown in Figure 2. The main characteristic absorption peaks and the corresponding groups are listed in Table 4.

**Figure 3:**
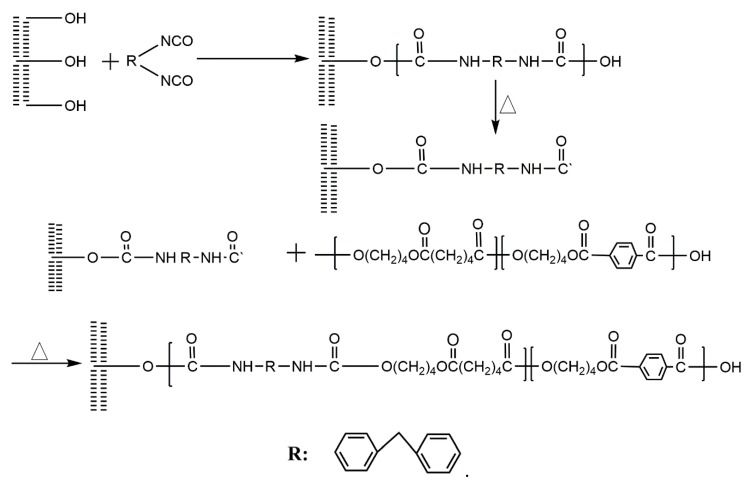
Reaction mechanism of BF/PBAT composites modified by MDI.

**Figure 4:**
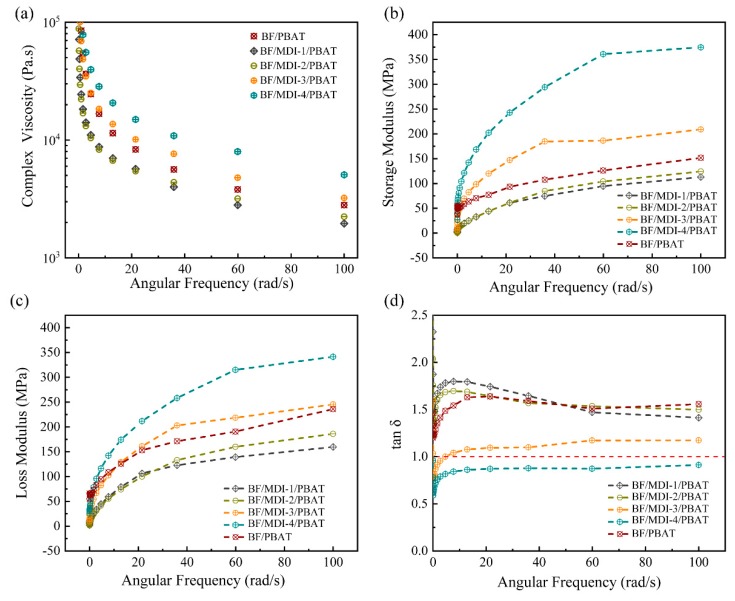
Effect of different contents of MDI on: (**a**) complex viscosity, (**b**) storage modulus, (**c**) loss modulus, and (**d**) damping factor.

For the BF/PBAT composites with MDI, a typical absorbance from carbamate (–NHCOO–) appeared at 1545 and 1636 cm^−1^. Meanwhile, the MDI characteristic peak of –N=C=O at 2250 cm^−1^ was not found from the spectra of composites. Furthermore, the disappearance of the –OH characteristic peak at 3500 cm^−1^ indicates that MDI reacts with –OH on the fiber surface. As a consequence, MDI is proven to be an effective compatibilizer to enhance the interfacial compatibility between bamboo powder and PBAT. The reaction mechanism of bamboo powder modified by MDI and PBAT are shown in Figure 3. The reaction mechanism between isocyanate and carboxyl groups are shown in Figure 4.

### 3.3. Rheological Properties of Composites

Rheological properties of BF/PBAT composites were analyzed with a rotational rheometer in order to determine the internal structure and dispersion state of bamboo powder in the composites. Figure 4 shows the complex viscosity, storage modulus, loss modulus, and loss factor of the BF/PBAT composites with different MDI contents. The complex viscosity is an important parameter to characterize the flow-processing performance of composite materials, and also can indirectly reflect the processing quality standard. As shown in Figure 4a, complex viscosity of all the composite melts decreased linearly with increasing angular frequency. This phenomenon is mainly attributed to the pseudoplastic shear thinning effect. The viscosity of the composites melt decreased in the following order: BF/MDI-4/PBAT > BF/MDI-3/PBAT > BF /PBAT > BF/MDI-2/PBAT > BF/MDI-1/PBAT. With the addition of MDI content, the complex viscosity initially decreased and then increased. This is primarily because of the decrease in interparticle interaction between bamboo particles after the surface hydroxyl groups’ reaction with MDI molecules. However, with the addition of MDI increased, the excessive MDI molecules could form a crosslink network between themselves, resulting in limiting the thermal motion of the matrix molecules, in turn increasing the modulus of the composite melts. 

The dynamic shear storage modulus (G’) and loss modulus (G”) of the composites as a function of frequency at 180 °C are shown in Figure 4b,c. The storage modulus refers to the elastic modulus, which reflects the elastic of the material, that is, rigidity, while the loss modulus refers to the viscosity modulus, which reflects the viscosity of the material, that is, creep. When the MDI content is 1 wt % and 2 wt %, the storage modulus and loss modulus of the composites are lower than that of BF/PBAT blend without MDI. As aforementioned, that is because of the decrease in interparticle interaction in the bamboo powder and the increase in the thermal motion of the molecules. When MDI content is higher than 3 wt %, the storage modulus were much higher than that of the BF/PBAT blend without the addition of MDI, indicating a development of a network structure in the composites.

The damping factor tan δ (= G”/G′) of the composites were shown in Figure 4d. In the whole frequency region, the tan δ of BF /PBAT, BF/MDI-1/PBAT and BF/MDI-2/PBAT is > 1.0, so that the loss modulus dominates the storage modulus. However, for BF/MDI-4/PBA, the tan δ is throughout < 1.0, indicating that the crosslinking network limits the mobility of polymer chains. As a result, the composites exhibited a greater storage modulus. 

### 3.4. Thermogravimetric Analysis of Composite

**Figure 5:**
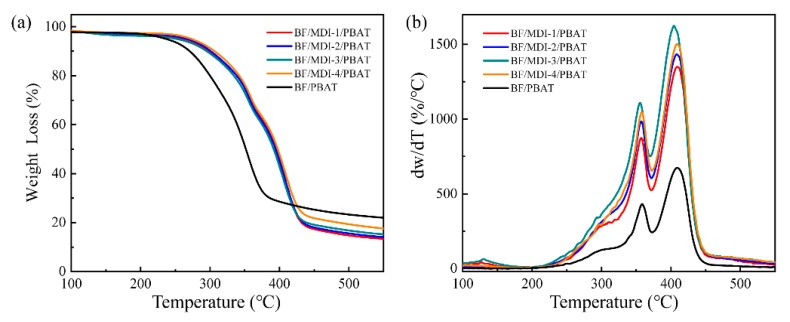
(**a**) TGA and (**b**) DTG curves of BF/PBAT composites without and with different contents of MDI.

In order to characterize the influence of diisocyanate on the thermal stability of the composites, thermogravimetric analysis was carried out. Figure 5 shows the TGA and derivative thermogravimetric (DTG) curves of the BF/PBAT composites without and with different content of MDI. The T_5 wt %_ and T_p_ temperatures of the composites are listed in Table 5. It was found that the T_5 wt %_ decomposition temperature of the BF/PBAT composite is 236.9 °C. After adding MDI, the T_5 wt %_ decomposition temperature of the composite increased from 236 to 268 °C, improving by 30 °C, indicating that addition of MDI can efficiently improve the thermal stability of the BF/PBAT composite. From Figure 5b, it can be seen that there are three peaks. The double peaks are due to the degradation of the BF/PBAT composite. The first is degradation of bamboo powder, about 356.7 °C, the second is degradation of PBAT, about 410.1 °C. The three peaks are due to the uneven molecular structure, which contains some small molecular substances. In the process of heating, some crosslinked small molecules are destroyed, leading to the loss of some raw materials. After adding a certain amount of diisocyanate, the temperature of the two thermal degradation processes almost has no obvious change. Because of the heterogeneity of molecular structure, the thermal degradation rate of *D_W_/D_T_* value of weight-loss rate changes greatly with the addition of MDI.

Because diisocyanate plays the role of chain extender in the mixing process, it not only increases the molecular weight, accelerates the thermal movement between molecules, but also increases the binding force between chemical bonds, making it not easy to be destroyed at high temperature. When diisocyanate is excessive, the initial thermal decomposition temperature and thermal degradation rate of diisocyanate decrease slightly due to the crosslinking effect of chemical bond.

### 3.5. Morphology of Fracture Surface

**Figure 6:**
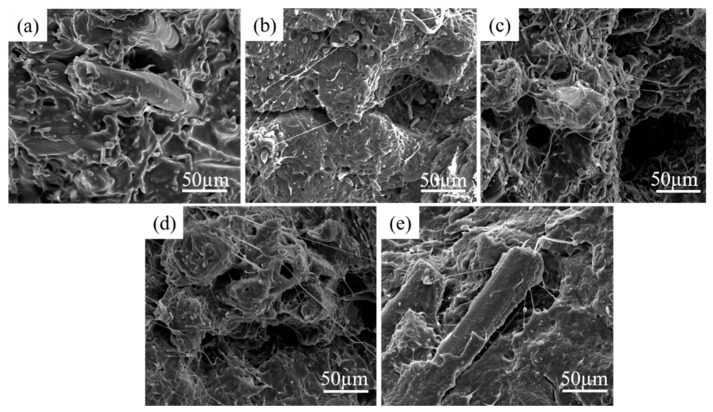
Fracture surface morphologies of: (**a**) BF/PBAT, (**b**) BF/MDI-1/PBAT, (**c**) BF/MDI-2/PBAT, (**d**) BF/MDI-3/PBAT and (**e**) BF/MDI-4/PBAT.

The SEM morphologies of impact fracture surface of BF/PBAT composites are shown in Figure 6. For BF/PBAT composites without compatibilizer, there are some cracks and voids between reinforcement and matrix, showing obviously roughness of interface (Figure 6a). This mainly because of the extraction of BF from PBAT during impact, resulting in stress concentration and defects, indicating poor adhesion and incompatibility between bamboo powder and the PBAT matrix. These defects may be the main reason for the decrease in mechanical properties of the bamboo plastic composite. Instead, the composites compatibilized by 1–3 wt % MDI (Figure 6b–d) have relatively continuous fracture surfaces. It should be noted that when the amount of MDI added is 4 wt % (Figure 6e), Figure 6a,e are almost identical. It is caused by excessive addition of diisocyanate and cross-linking.

Through the actions of MDI to expand the chain of PBAT, increase the molecular weight of PBAT, produce a cross-linking effect, form a strong interface layer, the stress acting on the matrix can be effectively transferred to BF. Figure 6 showed that when 2 wt % and 3 wt % MDI are added, the microstructure of the fiber changes significantly, and a large number of brushed MDI particles are attached to the fiber, so that the adhesion between the fiber and PBAT is enhanced. This is the result of toughness fracture of impact spline in the process of impact, which shows more toughness fracture of fiber. At this time, the microstructure is relatively flat and uniform, and the compatibility and adhesion are better. It shows that a certain amount of diisocyanate chain extender can improve the compatibility and impact strength of the composite. The results show that the fracture mode of the composite is fiber fracture. However, when the diisocyanate is added excessively, the drawstring fiber is obviously reduced, and the compatibility between the two phases is reduced. This is due to the crosslinking effect of MDI.

## 4. Conclusions

In summary, due to the hydrophobic nature of PBAT and hydrophilic nature of bamboo flour, a BF/PBAT (50/50) blend shows low mechanical properties, and especially shows poor impact strength. After adding reactive compatibilizer 4,4′-methylenebis (phenyl isocyanate) (MDI), BF/PBAT (50/50) composites with high mechanical properties were successfully prepared. The tensile strength, elongation at break, and impact strength of BF/MDI-2/PBAT composite with 2 wt % MDI content were increased by 1.9, 6.8, and 4.3 times respectively over those without the added MDI compatibilizer BF/PBAT blend. The higher toughening effect of MDI in BF/PBAT composites can be mainly ascribed to the improved interface bonding between BF and PBAT. The isocyanate group of MDI can react with the hydroxyl group of the BF surface, and in situ formation of the carbamate group on the BF surface. The residual isocyanate can then react with the hydroxyl group of PBAT and form carbamate groups. The rheology behaviors demonstrate that the addition of appropriate amounts of MDI, 1 wt % and 2 wt %, can promote the flowability of the resulting BF/PBAT composites due to the decrease in interparticle interaction between the bamboo powder, and the increase of the thermal motion of the molecules.

## Figures and Tables

**Table 1:** Physical properties and cost of natural fibers.

Material	Length (mm)	Diameter (µm)	Growth Cycle (month)	Cost ($)	Performance Advantages
Sisal [2]	1–1.5	20–25	24–36	14	High elasticity, great strength
Bagasse [5]	0.65–2.17	21–28	10–15	20	Good durability
Basalt fiber [10]	2.7–3	9–17	/	2–18	High strength and high temperature resistance
Wood flour [7]	60–200	8–12	12	20–30	Good flexibility and moisture resistance
Bamboo fiber [3]	60–200	6–10	3–6	1.3–2	Antibacterial, bacteriostatic deodorant, UV resistant
Banana fiber [11]	70	18–24	10–15	10–12	Lightweight, good luster, high water absorption, and strong antibacterial

**Table 2:** Mixing ratio of composite materials.

Sample Codes	BF (wt %)	PBAT (wt %)	MDI/phr
BF/PBAT	50	50	0
BF/MDI-1/PBAT	50	50	1
BF/MDI-2/PBAT	50	50	2
BF/MDI-3/PBAT	50	50	3
BF/MDI-4/PBAT	50	50	4

**Table 3:** Mechanical properties of bamboo flour/ poly (butylene terephthalate) (BF/PBAT) composites.

Sample Codes	Tensile Strength (MPa)	Tensile Modulus (Pa)	Impact Strength (KJ/m^2^)	Elongation at Break (%)
BF/PBAT	15 ± 1.5	693.8 ± 50	6.7 ± 0.8	5.6 ± 0.5
BF/MDI-1/PBAT	26.2 ± 0.8	1021.5 ± 120	36.6 ± 1.5	16.3 ± 0.3
BF/MDI-2/PBAT	28.6 ± 1.6	1292.6 ± 80	45.6 ± 3.8	24.2 ± 0.4
BF/MDI-3/PBAT	27.6 ± 1.8	1208.7 ± 110	51.2 ± 7.5	22.7 ± 1.8
BF/MDI-4/PBAT	27.3 ± 2.1	1105.4 ± 80	48.7 ± 7.8	22 ± 1.3

**Table 4:** Wave number of characteristic functional group.

Wavenumber (cm^−1^)	Assignment
3500–3100	N–H stretching
2270–2000	–N=C=O asymmetric stretching
1720–1640	C=O stretching
1600	C=C aromatic stretching
1535–1520	–HNCO– symmetric stretching
1230–1250	C–N stretching

**Table 5:** Thermal weight-loss data of different content of diisocyanate on composites.

Sample Codes	T_5 wt %_ (°C)	T_p_ (°C)	
		BF	PBAT
BF/PBAT	236.9	356.7	410.1
BF/MDI-1/PBAT	264.0	357.1	409.2
BF/MDI-2/PBAT	266.4	357.4	408.2
BF/MDI-3/PBAT	267.6	356.8	404.0
BF/MDI-4/PBAT	266.6	358.0	408.7

## References

[1] Raja D.B.P., Retnam B.S.J. (2019). Effect of short fibre orientation on the mechanical characterization of a composite material-hybrid fibre reinforced polymer matrix. Bull. Mater. Sci..

[2] Uppal N., Pappu A., Patidar R., Gowri V.S. (2019). Synthesis and characterization of short sisal fibre polyester composites. Bull. Mater. Sci..

[3] Long H., Wu Z., Dong Q., Shen Y., Zhou W., Luo Y., Zhang C., Dong X. (2019). Effect of polyethylene glycol on mechanical properties of bamboo fiber-reinforced polylactic acid composites. J. Appl. Polym. Sci..

[4] Kim H.-S., Kim H.-J. (2013). Miscibility and performance evaluation of natural-flour-filled PP/PBS and PP/PLA bio-composites. Fibers Polym..

[5] Oladele I.O., Ibrahim I.O., Akinwekomi A.D., Talabil S.I. (2019). Effect of mercerization on the mechanical and thermal response of hybrid bagasse fiber/CaCO3 reinforced polypropylene composites. Polym. Test..

[6] Woigk W., Fuentes C.A., Rion J., Hegemann D., van Vuure A.W., Dransfeld C., Masania K. (2019). Interface properties and their effect on the mechanical performance of flax fibre thermoplastic composites. Compos. Part A Appl. Sci. Manuf..

[7] Yao L., Wang Y., Li Y., Duan J. (2017). Thermal properties and crystallization behaviors of polylactide/redwood flour or bamboo fiber composites. Iran. Polym. J..

[8] Jandas P.J., Mohanty S., Nayak S.K. (2013). Thermal properties and cold crystallization kinetics of surface-treated banana fiber (BF)-reinforced poly(lactic acid) (PLA) nanocomposites. J. Therm. Anal. Calorim..

[9] Jandas P.J., Mohanty S., Nayak S.K. (2013). Mechanical properties of surface-treated banana fiber/polylactic acid biocomposites: A comparative study of theoretical and experimental values. J. Appl. Polym. Sci..

[10] Liu T., Yu F., Yu X., Zhao X., Lu A., Wang J. (2012). Basalt fiber reinforced and elastomer toughened polylactide composites: Mechanical properties, rheology, crystallization, and morphology. J. Appl. Polym. Sci..

[11] Jandas P.J., Mohanty S., Nayak S.K., Srivastava H. (2011). Effect of Surface Treatments of Banana Fiber on Mechanical, Thermal, and Biodegradability Properties of PLA/Banana Fiber Biocomposites. Polym. Compos..

[12] Fazeli M., Florez J.P., Simao R.A. (2019). Improvement in adhesion of cellulose fibers to the thermoplastic starch matrix by plasma treatment modification. Compos. Part B Eng..

[13] Su S.-K., Wu C.-S. (2010). The Processing and Characterization of Polyester/Natural Fiber Composites. Polym. Plast. Technol. Eng..

[14] Wang Y.-N., Weng Y.-X., Wang L. (2014). Characterization of interfacial compatibility of polylactic acid and bamboo flour (PLA/BF) in biocomposites. Polym. Test..

[15] Song X.-Y., Wang M., Weng Y.-X., Huang Z.-G. (2017). Effect of Bamboo Flour Grafted Lactide on the Interfacial Compatibility of Polylactic Acid/Bamboo Flour Composites. Polymers.

[16] Sun J., Pang Y., Yang Y., Zhao J., Xia R., Li Y., Liu Y., Guo H. (2019). Improvement of Rice Husk/HDPE Bio-Composites Interfacial properties by Silane Coupling Agent and Compatibilizer Complementary Modification. Polymers.

[17] Kabir M.M., Wang H., Lau K.T., Cardona F. (2012). Chemical treatments on plant-based natural fibre reinforced polymer composites: An overview. Compos. Part B Eng..

[18] Quiles-Carrillo L., Boronat T., Montanes N., Balart R., Torres-Giner S. (2019). Injection-molded parts of fully bio-based polyamide 1010 strengthened with waste derived slate fibers pretreated with glycidyl-and amino-silane coupling agents. Polym. Test..

[19] Safri S.N.A., Sultan M.T.H., Saba N., Jawaid M. (2018). Effect of benzoyl treatment on flexural and compressive properties of sugar palm/glass fibres/epoxy hybrid composites. Polym. Test..

[20] Wang D., Bai T., Cheng W., Xu C., Wang G., Cheng H., Han G. (2019). Surface Modification of Bamboo Fibers to Enhance the Interfacial Adhesion of Epoxy Resin-Based Composites Prepared by Resin Transfer Molding. Polymers.

[21] Fan W., Tian H., Wang H., Zhang T., Yang X., Yu Y., Meng X., Yu X., Yuan L., Xu B. (2018). Enhanced interfacial adhesion of aramid fiber III reinforced epoxy composites via low temperature plasma treatment. Polym. Test..

[22] Liew F.K., Hamdan S., Rahman M.R., Mahmood M.R., Lai J.C.H. (2018). The effects of nanoclay and tin(IV) oxide nanopowder on morphological, thermo-mechanical properties of hexamethylene diisocyanate treated jute/bamboo/polyethylene hybrid composites. J. Vinyl Addit. Technol..

[23] Ye M., Zhu N.Q., Li X.J., Ni Z.B., Qiu Z.W., Chen M.Q. (2017). Monofunctional compatibilizer with long alkyl end for fabrication of superior tensile wood flour-polyolefin composites. J. Appl. Polym. Sci..

[24] Abushammala H., Mao J. (2019). A Review of the Surface Modification of Cellulose and Nanocellulose Using Aliphatic and Aromatic Mono-and Di-Isocyanates. Molecules.

[25] Guo C., Li L., Li H. (2019). Evaluation of interfacial compatibility in wood flour/polypropylene composites by grafting isocyanate silane coupling agent on polypropylene. J. Adhes. Sci. Technol..

[26] Saeng-on J., Aht-Ong D. (2018). Compatibility of banana starch nanocrystals/poly(butylene succinate) bio-nanocomposite packaging films. J. Appl. Polym. Sci..

[27] Chan C.M., Vandi L.J., Pratt S., Halley P., Richardson D., Werker A., Laycock B. (2018). Mechanical properties of poly(3-hydroxybutyrate-*co*-3-hydroxyvalerate)/wood flour composites: Effect of interface modifiers. J. Appl. Polym. Sci..

[28] Rahimizadeh A., Kalman J., Henri R., Fayazbakhsh K., Lessard L. (2019). Recycled Glass Fiber Composites from Wind Turbine Waste for 3D Printing Feedstock: Effects of Fiber Content and Interface on Mechanical Performance. Materials.

[29] Soundhar A., Jayakrishna K. (2019). Investigations on mechanical and morphological characterization of chitosan reinforced polymer nanocomposites. Mater. Res. Express.

[30] Huang G., Chen F. (2019). Reaction of jute fiber with isocyanate component for the production of plant fiber-reinforced polyurethane composites. Cellulose.

[31] Tayfun U., Dogan M., Bayramli E. (2017). Investigations of the flax fiber/thermoplastic polyurethane eco-composites: Influence of isocyanate modification of flax fiber surface. Polym. Compos..

